# Awareness, attitudes and prevention of malaria in the cities of Douala and Yaoundé (Cameroon)

**DOI:** 10.1186/1756-3305-4-181

**Published:** 2011-09-20

**Authors:** Cyrille Ndo, Benjamin Menze-Djantio, Christophe Antonio-Nkondjio

**Affiliations:** 1 Laboratoire de Recherche sur le Paludisme, Organisation de Coordination pour la lutte Contre les Endémies en Afrique Centrale (OCEAC), P.O. Box 288, Yaoundé, Cameroon; 2 Faculty of Sciences, University of Yaoundé I, P.O. Box 337, Yaoundé, Cameroon; 3 Vector group, Liverpool School of Tropical Medicine, Pembroke Place, Liverpool L3 5QA, UK

**Keywords:** Malaria prevention, awareness, knowledge, households, Yaoundé, Douala

## Abstract

**Background:**

There is little information on the social perception of malaria and the use of prevention methods in Cameroon. This study was designed to assess knowledge, attitude and management of malaria in households living in the cities of Douala and Yaoundé.

**Results:**

Over 82% of people interviewed associated malaria transmission to mosquito bites. Methods used for malaria prevention were: environmental sanitation 1645 (76.1%), use of bed nets 1491 (69%), insecticide spray/coils 265 (12.3%) and netting of doors or windows 42 (1.9%). Bed net ownership was significantly high in Yaoundé (73.8%) (P < 0.0001), whereas the use of insecticide spray or coils was significantly important in Douala (16.3%) (P < 0.0001). Some of the problems experienced by families using ITN were the difficulty in finding chemicals for the retreatment of nets 702 (47%), insufficient financial means to buy new bed nets to replace old ones 366 (24.5%) or, to provide bed nets to everybody in the household 289 (19.4%) and the sensation of feeling excessive heat when sleeping under a bed net 74 (5%). The amount spent monthly by a household for vector control and malaria treatment was estimated at 2377 fcfa (3.6 euros) and 4562 fcfa (6.95 euros) respectively. These amounts were not significantly different between households of Douala and Yaoundé. Concerning management of malaria cases, 18.6% of people declare going to the hospital when suffering from malaria. The majority of people (81.4%) do self medication - they either buy drugs from the pharmacists, street sellers or they use plants to cure malaria.

**Conclusion:**

The study revealed a high awareness of populations on malaria and ITNs. However some attitudes hindering the use of ITN or related to the management of clinical cases need further attention.

## Background

Malaria remains a major public health threat, particularly in Africa where over 90% of cases are recorded [1]. It is estimated that 250 million cases and 880 000 deaths occur worldwide yearly [1]. Malaria has generally been considered as a disease of rural areas, but many factors linked to a rapid and uncontrolled urbanization are increasing malaria transmission in cities across Africa [2,3]. The expansion of malaria transmission to urban areas is of particular concern to malaria control programs since populations in these areas are likely to be at higher risk of the development of severe malaria due to the lack of protective immunity. In Cameroon, the disease is annually responsible for 30 to 35% of the total deaths, 35% of childhood mortality and 40 to 45% of morbidity cases [4]. According to recent records from the Cameroon Ministry of Health, it is estimated that 41% of the population has at least one episode of malaria each year, children under 5 and pregnant women being the most affected [4]. With a total population of about 20 million, this represents 8.2 million people suffering from malaria attacks each year. Current malaria control strategies in the country consist of the diagnosis and treatment of clinical cases and the promotion of ITNs. The use of ITNs is among the major recommendations for malaria control under the Roll Back Malaria initiative [5]. To achieve its goals of reducing the burden of malaria in the country, the government has taken since 2003, several initiatives. Measures taken include (i) the free distribution of ITNs to pregnant women and children under five years old, (ii) the subsidizing of the cost of ITN for other people, (iii) the subsidizing of the cost of artemisinin-based combination therapy use as first line treatment for uncomplicated malaria cases and, (iv) the training in the community of local health assistants capable of managing uncomplicated malaria cases and providing adequate advices to families [4]. With these set of measures, the Cameroon government expects noticeable changes on the prevalence and the control of the disease. Over 53% of the Cameroon population live in towns the number is increasing each year [6]. Data on the quantitative assessment of malaria burden in Cameroon cities and the analysis of community attitudes and knowledge on malaria are currently lacking and could help to better design and monitor malaria control programs on the field. Assessing the knowledge of communities is of paramount importance for the adoption of good practices for health improvement and disease prevention. This study was designed to assess the general knowledge and identify the attitudes of inhabitants of the cities of Douala and Yaoundé on the prevention and home management of malaria cases.

## Material and methods

The study was conducted in the cities of Yaoundé (3°51'N 11°30'E) and Douala (3°48'N 10°08'E) the two major urban cities in Cameroon with each having over 2 million inhabitants. These cities are situated within the Congo-Guinean phytogeographic zone characterized by a typical equatorial climate with two rainy seasons extending from March to June and from September to November. Douala is situated near the Atlantic coast 1 m above sea level. Yaoundé on the other hand, is located inland 250 km east of Douala. The city is situated 800 m above sea level and surrounded by many hills. Malaria transmission in these two cities is considered holoendemic and seasonal with *Anopheles gambiae *as the main vector [7]. Average annual prevalence of *Plasmodium falciparum *in the general population is estimated to vary between 34% to 50% from the city centre to the periphery. The age group 0-15 years is considered the most affected and comprised 75% of asexual parasites carriers, 85% of carriers of high parasitaemias and 83% of gametocyte carriers [8,9].

Investigations in the city of Yaoundé were conducted in fifteen districts: Ahala, Biyem-Assi, Mballa 2, Melen, Messa, Mokolo, Mvogada, Etoug-Ebe, Mvogbeti, Mvogbi, Nkolbisson, Nkomkana, Obili, Tsinga and Nkolkumu. In Douala, investigations took place in fourteen districts: Bonaberi, Bessengue, Beedi, Bonamoussadi, Bonangang, Cité des palmiers, Nylon, Ndogbong, Dakar, Makepé, Malangue, New-Bell, PK8, Village. Districts selected were distributed from the periphery to the city centre and included highly populated, well urbanized, and spontaneously urbanized districts.

The study was conducted from October 2009 to June 2010. Parents (household head or his spouse) who consented to the study, were interviewed using a structured pre-tested questionnaire. Interviews were conducted in private to reduce the influence from other people. Interviews were conducted in French or English. A questionnaire consisting of 10 questions was administered to know if: (i) people are aware of the causes of malaria, (ii) they know clinical symptoms of malaria (iii) they have any knowledge about mosquito breeding sites, (iv) they use any prevention method for vector control, (v) the origin of their bed nets (vi) estimate the amount spent monthly (in Franc CFA (FCFA) the local currency) for prevention and the treatment of malaria and (vii) how they manage malaria cases at home. The study was conducted under the ethical clearance N°216/CNE/SE/09 delivered by the Cameroon National Ethics Committee Ref N°IORG0006538-IRB00007847-FWA00016054.

### Data analysis

Once recorded, the data were entered in an Excel database and analyzed using the software EPI-INFO V3.5.2. and MedCalc V11.5.0.0. Percentages were compared using chi squared test or Fisher's exact test. Comparison between means was assessed using ANOVA or Kruskal Wallis test in case of inequality of sample variances.

A basic socio-economic indicator was developed based on information on the type of house owned. Habitats constructed with mud, cemented walls or plank with poor facilities for hygiene had the lowest indicator. Houses constructed with brick and cement equipped with all the necessary facilities for a good living standard had the highest indicator.

## Results

### Socio-demographic characteristics

A total of 2161 households out of 2403 contacted agreed to join the study representing a participation rate of 90%. The total number of households interviewed per city was 1053 in Douala and 1108 in Yaoundé. Households interviewed included families from all socio-economic classes (low, medium and high income classes). The average number of persons and children per household was 5.4 and 3 respectively.

### General Knowledge on malaria and its vector

Almost all parents interviewed 1972 (91.2%) associated malaria with fever. The majority of people interviewed in Douala 799 (75.9%) and in Yaoundé 977 (88.2%) knew that mosquitoes were responsible for malaria transmission. The remaining people (17.8%) attributed disease transmission to various factors (witchcraft, consumption of dirty water, cold weather...). Of the 2161 households interviewed, 1713 (79.3%) knew mosquito breeding habitats (Table 1).

**Table 1:** Characteristics, knowledge and level of expenses for vector control and malaria treatment of households interviewed in Douala and Yaoundé

Categories	Characteristics	Douala	Yaoundé	Both cities
Households	N interviewed	1053	1108	2161
	N households/district	81	79	80
	Mean person/household	5.2 ± 0.18	5.5 ± 0.16	5.37 ± 0.13
Cause of malaria	Mosquitoes bites	799 (75.9%)	977 (88.2%)	1776 (82.2%)
	Other (witchcraft, dirty water, cold weather)	254 (24.1%)	131 (11.8%)	385 (17.8%)
Breeding sites	Know	784 (74.4%)	929 (83.8%)	1713 (79.4%)
	Don't know	268 (25.6%)	177 (16.2%)	445 (20.6%)
Prevention	Environmental sanitation	821 (78%)	824 (74.4%)	1645 (76.1%)
	Use of bed nets	673 (64%)	818 (73.8%)	1491 (69%)
	Insecticide spray/coils	172 (16.3%)	93 (8.4%)	265 (12.3%)
	Netting (doors or windows)	11 (1%)	31 (2.8%)	42 (1.9%)
	No preventive measure	197 (18.7%)	165 (14.9%)	362 (16.7%)
Problems of ITNs users	Retreatment of expired ITNs	280 (41.6%)	422 (51.6%)	702 (47%)
	Feel hot under ITNs	43 (6.4%)	31 (3.8%)	74 (5%)
	No financial means to replace old nets	196 (29.1%)	170 (20.8%)	366 (24.5%)
	No financial means to provide nets to the family	139 (20.6%)	150 (18.3%)	289 (19.4%)
Origin of Bed nets	Freely acquired	231 (34.3%)	289 (35.3%)	520 (34.9%)
	Bought	442 (65.7%)	529 (64.7%)	971 (65.1%)
Expenses (FCFA)	For mosquito control	2266 ± 230 (3.4 Euros)	2507 ± 413 (3.8 Euros)	2377.38 ± 187 (3,6 Euros)
	For malaria treatment	4652 ± 352 (7.09 Euros)	4472 ± 544 (6.8 Euros)	4562 ± 322 (6.95 Euros)

Methods used for malaria prevention were: environmental sanitation 1645 (76.1%), the use of bed nets 1491 (69%), insecticide spray/coils 265 (12.3%) and netting of doors or windows 42 (1.9%) (Table 1). The proportion of households owning bed nets in Yaoundé (73.8%) was significantly important than in Douala (64%) (χ^2 ^= 24.3; df = 1 P < 0.0001). Bed net ownership was defined as families having at least one bed net. About one quarter of families owning bed nets in Douala (24.4%) and 16.9% in Yaoundé, declared using in addition, insecticide spray or coils. The possession of bed nets in both cities varied greatly from one district to another. In Yaoundé, high frequencies of bed nets ownership (> 70% of families interviewed) were recorded in the districts of Mokolo, Mvog-Mbi, Biyem-Assi, Ngousso, Mballa 2 and Nkolbisson. In Douala, a high rate of bed net ownership was recorded in the districts of Village and Ndogbong (> 70% of families interviewed). Of the 1491 households who declared owning a bed net, 520 (34.9%) declared having acquired bed nets from the free distribution campaign organized by the Ministry of Health. The remaining others (65.1%) bought theirs. The proportion of households having acquired free bed nets was not significantly different between the two cities (P = 0.98).

The proportion of families using insecticide spray or coils was significantly important in Douala (16.3%) compare to Yaoundé (8.4%) (χ^2 ^= 30.9, df = 1, P < 0.0001). Insecticide spray or coils were more frequently used by families residing in the districts of Malangue, Bonaberi, PK8, Bessengue, Cité des Palmiers in Douala and Messa in Yaoundé (> 30% of persons interviewed). Besides, 362 (16.7%) households (197 in Douala and 165 in Yaoundé) declared not using any protection measure. The majority of these families were residents of well urbanized areas or of districts situated in the city centre.

Some of the problems experienced by families using ITN were the difficulty to find chemicals for retreatment of nets 702 (47%), insufficient financial means to buy new bed nets to replace old ones 366 (24.5%) or, to provide bed nets to everybody in the household 289 (19.4%) and the sensation of feeling excessive heat when sleeping under a bed net 74 (5%) (Table 1).

### Financial cost associated with vector control and malaria treatment in surveyed households

Expenses for vector control were particularly important for households living in poorly urbanized districts or near swamps, lakes or rivers. In these areas, families declared spending between 5 to 15% of their monthly revenues for vector control. For malaria treatment, the amount spent could go up to 30% of the monthly revenues depending on the seriousness of the case. In average, the amount spent monthly by a family was 2377 fcfa (3.6 euros) and 4562 fcfa (6.95 euros) for mosquito burden and malaria treatment respectively (Table 1). These amounts were not significantly different between Douala and Yaoundé (P > 0.29).

### Home management of malaria cases

About 9% (n = 96) and 19% (n = 206) of the people interviewed in Douala and Yaoundé respectively, declared going to the hospital when they or one of their relative have malaria (Table 2). These proportions were significantly different between the two cities (p < 0.0001). People declared doing the following when they are ill: buying drugs from the pharmacy 769 (35.9%), to street sellers 481 (22.4%), buying drugs either from the pharmacy or on the streets 118 (5.5%), using traditional medicine (plants) 76 (3.5%). Some adults declared combining drugs bought from the pharmacy or on the streets with traditional medicine (plants) 397 (18.5%) particularly during severe malaria episodes (Table 2). For the majority of people (> 80% of respondents), going to the hospital appears as the ultimate solution when self- medication has failed. Reliance on the drugs sold on the street was similar for the two cities. The reason for buying these drugs was mainly associated with the differences in the costs.

**Table 2:** Home management of malaria cases by households in the cities of Douala and Yaoundé

Characteristics	Douala (%)	Yaoundé (%)	Both cities (%)
N respondents	1039	1104	2143
Hospital	96 (9%)	206 (19%)	302 (14%)
Pharmacy	377 (36.3%)	392 (35.5%)	769 (35.9%)
Street drugs	216 (20.8%)	265 (24%)	481 (22.4%)
Plants (traditional medicine)	34 (3.3%)	42 (3.8%)	76 (3.5%)
Pharmacy & street drugs	58 (5.6%)	60 (5.4%)	118 (5.5%)
Pharmacy, street drugs and plants	258 (24.8%)	139 (12.6%)	397 (18.5%)

## Discussion

The majority of people interviewed knew malaria prevention methods. Four preventive measures were used by urban dwellers in Douala and Yaoundé, environmental sanitation, bed nets, insecticide spray or coils, and netting at doors or windows. Over 60% of people interviewed declared using impregnated bed nets to prevent malaria. Indeed malaria control strategy in Cameroon mainly relies on the use of ITNs. For several years, this strategy is benefiting from mass campaign promotion and is advertised through audiovisual networks and newspapers. This could explain the increased utilization of this method compare to previous years [10]. In addition to promotion campaigns, over 2 million impregnated bed nets have been freely distributed to pregnant women and children under 5 years across the country. However despite the increased ownership of bed nets, a decrease in malaria transmission or morbidity has not been reported [11]. This suggests that ownership of impregnated bed nets has not enhanced their utilization. This could be linked to a good number of reasons raised in previous studies conducted on the continent such as: social behaviors of communities, size and type of the house, level of education, ethnicity, seasons, frequency of retreatment of nets [12-14]. When properly used, ITNs can reduce by 50% the rate of malaria transmission and subsequent morbidity and mortality [15,16]. Among practices hindering the use of bed nets in the cities of Douala and Yaoundé were the sensation of discomfort when sleeping under bed net as expressed by some users living in densely populated areas. The insufficient financial means to buy new nets to replace old nets or to provide nets to everybody especially in large size families was also reported. The lack of financial means as a source of non-use of bed nets has been reported in several studies [17,18]. Another important point is the retreatment of bed nets with chemicals which is rarely done. To overcome this problem, the strategy adopted by many African countries is to switch to long lasting nets [19-22]. This strategy has been adopted by the Cameroon government which intends to distribute several millions of long lasting nets across the country following it admission to the Global Fund initiative [4]. The proportion of people owning impregnated bed nets was more important in the city of Yaoundé than in Douala and could have been favored by the temperate climate in Yaoundé a town situated at altitude compare to Douala situated at sea level. Although important, ITNs implementation in the cities of Douala and Yaoundé could be particularly ineffective due to night activities or leisure keeping people very late outside. Moreover the use of ITNs could be seriously jeopardized with the increased prevalence of vector resistance to insecticide [23]. These drawbacks to the use of ITNs in these urban areas, suggest that ITNs should be complimented with other control measures such as larval control especially in high malaria transmission risk areas [24].

The amount spent by families to fight mosquito burden and malaria treatment was more important than amounts reported by previous studies two decades before [25]. These observations are consistent with the high malaria burden in these cities characterized by annual prevalence of malaria parasite in the general population varying from 35 to 50% and high parasitaemias (> 400 parasites/μl of blood) particularly in the age group of 0 to 15 years [8,9]. Over 80% of people interviewed declared practicing self medication. The recourse to pharmacy and street drugs is favored by the fact that in Cameroon, people do not need any medical prescription to get drugs from pharmacists. Drugs sold on the street are suspected to be of low quality due to the absence of any control of quality for these drugs which are locally manufactured or imported illegally. More than 20% of people interviewed buy drugs from the street. For the cities of Yaoundé and Douala who have over 2 000 000 inhabitants each, this represent over 400 000 people in each city using these drugs. The situation needs further attention in order to improve the quality of services delivered to the population and to reduce the expansion of drug resistance.

## Conclusion

The study showed, despite a high awareness, a low utilization of ITNs for malaria prevention supporting the need for regular educational campaigns to increase bed net use and to promote environmental sanitation measures in communities. The current expansion of illegal drugs business, require proper control measures by the government to insure that, good quality health care services are delivered to the population.

## Competing interests

The authors declare that they have no competing interests.

## Authors' contributions

Conceived and designed the study protocol: CAN, CN Participated to field investigations, BMD, CN, CAN. Interpreted, analysed data and wrote the manuscript: CAN. All authors read and approved the final version of the manuscript.

## References

[B1] WHO World Malaria Report 2009 2009 http://www.who.int/malaria/world_malaria_report_2009/en/

[B2] Keiser J Utzinger J Caldas de Castro M Smith T Tanner M Singer B Urbanization in sub-saharan Africa and implication for malaria control Am J Trop Med Hyg 2004 71 118 127 15331827

[B3] Wang S Lengeler C Smith T Vounatsou P Cisse G Diallo D Akogbeto M Mtasiwa D Teklehaimanot A Tanner M Rapid urban malaria appraisal (RUMA) in sub-Saharan Africa Malar J 2005 4 40 10.1186/1475-2875-4-40 16153298 PMC1249588

[B4] Minsanté Cameroun: vers un meilleur accès à la prévention et au traitement du SIDA, de la tuberculose et du paludisme 2008 http://www.afriscoopnet/journal/spipphp

[B5] WHO The African summit on Roll Back Malaria WHO/CDS/RBM/2000-1722000 2000 World Health Organization, Geneva

[B6] BUCREP Troisième recencement générale de la population et de l'habitat. Third general population and housing census Cameroun Rapport de présentation des résulstats définitifs République du Cameroun 2010 1 65

[B7] Craig M Snow R le Sueur D A climate-based distribution model of malaria transmssion in sub-Saharan Africa Parasitol Today 1999 15 3 105 111 10.1016/S0169-4758(99)01396-4 10322323

[B8] Van der Kolk M Etti Tebo A Nimpaye H Ngo Ndombol D Sauerwein R Eling W Transmission of Plasmodium falciparum in urban Yaoundé Cameroon is seasonal and age-dependent Trans R Soc Trop Med and Hyg 2003 97 375 379 10.1016/S0035-9203(03)90059-9 15259460

[B9] Quakyi I Leke R Befidi-Mengue R Tsafack M Bomba-Nkolo D Manga L Tchinda V Njeungue E Kouontchou S Fogako J The epidemiology of Plasmodium falciparum malaria in two Cameroonian villages: Simbok and Etoa Am J Trop Med Hyg 2000 63 222 230 11421368

[B10] Etang J Fondjo E Bintsindou P Bagayoko M Manga L Profil entomologique du Cameroun Rapport Minsanté Cameroun et OMS 2007 1 4221931042

[B11] Minsanté Rapport sur l'évolution des cas d'accès palustres dans les districts de santé de la ville de Yaoundé Rapport du Programme National de lutte contre le paludisme du Cameroun 2010 35

[B12] Eisele T Keating J Littrel M Larsen D McIntyre K Assessment of Insecticide-Treated Bednet use among children and pregnant women across 15 countries using standardized national surveys Am J Trop Med Hyg 2009 8 209 214 19190215

[B13] Toe L Skovmand O Dabiré K Diabate A Diallo Y Guiguemde R Doannio C Akogbeto M Baldet T Gruénais E Decreased motivation in the use of insecticide-treated nets in a malaria endemic area in Burkina Faso Malar J 2009 8 175 10.1186/1475-2875-8-175 19640290 PMC2729312

[B14] Atieli H Zhou G Afrane Y Lee M-C Mwanzo I Githeko A Yan G Insecticide-treated net (ITN) ownership, usage, and malaria transmission in the highlands of western Kenya Parasites & Vectors 2011 4 1 113 10.1186/1756-3305-4-113 21682919 PMC3135563

[B15] D'Alessandro U Olaleye B McGuire W Langerock P Bennett S Aikins M Thomson M Cham B Greenwood B Mortality and morbidity from malaria in Gambian children after introduction of a treated bednets programme The Lancet 1995 345 479 483 10.1016/S0140-6736(95)90582-0 7861874

[B16] Nevill C Some E Mung'ala V Mutemi W New L Marsh L Lengeler C Snow R Insecticide-treated bednets reduce mortality and severe morbidity from malaria among children on the Kenyan coast Trop Med Int Health 1996 1 139 146 8665377 10.1111/j.1365-3156.1996.tb00019.x

[B17] Schellenberg J Abdulla S Nathan R Mukasa O Marchant T Kikumbih N Mushi A Mponda H Minja H Mshinda H Effect of large-scale social marketing of insecticide-treated nets on child survival in rural Tanzania Lancet 2001 357 1241 1247 10.1016/S0140-6736(00)04404-4 11418148

[B18] Musa O Salaudeen G Jimoh R Awareness and use of insecticide treated nets among women attending ante-natal clinic in a northern state of Nigeria J Park Med Assoc 2009 56 354 358 19534367

[B19] Thwing J Perry R Townes D Diouf M Ndiaye S Thior M Success of Senegal's first nationwide distribution of long-lasting insecticide-treated nets to children under five contribution toward universal coverage Malar J 2011 10 86 10.1186/1475-2875-10-86 21489278 PMC3083382

[B20] Bonner K Mwita A McElroy P Omari S Mzava A Lengeler C Kaspar N Nathan R Ngegba J Mtung'e R Design, implementation and evaluation of a national campaign to distribute nine million free LLINs to children under five years of age in Tanzania Malar J 2011 10 73 10.1186/1475-2875-10-73 21453519 PMC3078903

[B21] Koudou B Ghattas H Esse C Nsanzabana C Rohner F Utzinger J Faragher B Tschannen A The use of insecticide-treated nets for reducing malaria morbidity among children aged 6-59 months, in an area of high malaria transmission in central Cote d'Ivoire Parasites & Vectors 2010 3 1 91 10.1186/1756-3305-3-91 20860829 PMC2955709

[B22] Chandre F Dabire R Hougard J-M Djogbenou L Irish S Rowland M N'Guessan R Field efficacy of pyrethroid treated plastic sheeting (durable lining) in combination with long lasting insecticidal nets against malaria vectors Parasites & Vectors 2010 3 1 65 10.1186/1756-3305-3-65 20682050 PMC2920241

[B23] Antonio-Nkondjio C Tene-Fossog B Ndo C Menze-Djantio B Zebaze-Togouet S Awono-Ambene H Costantini C Wondji C Ranson H Anopheles gambiae distribution and insecticide resistance in the cities of Douala and Yaoundé (Cameroon): influence of urban agriculture and pollution Malar J 2011 10 154 10.1186/1475-2875-10-154 21651761 PMC3118161

[B24] Imbahale S Mweresa C Takken W Mukabana W Development of environmental tools for anopheline larval control Parasites & Vectors 2011 4 1 130 10.1186/1756-3305-4-130 21733150 PMC3143094

[B25] Louis J Trebucq A Gelas H Fondjo E Manga L Toto J Carnevale P Le paludisme maladie dans la ville de Yaoundé (Cameroun). Prise en charge et lutte antivectorielle au niveau familial Bull Soc Path Exoth 1992 85 26 30 1596954

